# Determinants of Antenatal Care Utilization Among Childbearing Women in Burkina Faso

**DOI:** 10.3389/fgwh.2022.848401

**Published:** 2022-05-24

**Authors:** Hermann Badolo, Aristide Romaric Bado, Hervé Hien, Manuela De Allegri, Appunni Sathiya Susuman

**Affiliations:** ^1^Department of Statistics and Population Studies, Faculty of Natural Sciences, University of the Western Cape, Cape Town, South Africa; ^2^Centre MURAZ, Institut National de Santé Publique, Ouagadougou, Burkina Faso; ^3^Département Biomedical et Santé Publique, Institut de Recherche en Sciences de la Santé, Ouagadougou, Burkina Faso; ^4^West African Health Organization, Bobo-Dioulasso, Burkina Faso; ^5^Département Biomedical et Santé Publique, Institut de Recherche en Sciences de la Santé, Bobo-Dioulasso, Burkina Faso; ^6^Heidelberg Institute of Global Health, Heidelberg University Hospital and Medical Faculty, Heidelberg University, Heidelberg, Germany

**Keywords:** antenatal care, multilevel analysis, determinants, health services utilization, Burkina Faso

## Abstract

**Introduction:**

Antenatal care (ANC) is one of the pillars of maternal and child health programs aimed at preventing and reducing maternal and child morbidity and mortality. This study aims to identify the factors associated with ANC use, considering both health care demand and supply factors in the single analysis.

**Methods:**

We used data from the endline survey conducted to evaluate the impact of the performance-based financing (PBF) program in Burkina Faso in 2017. This study was a blocked-by-region cluster random trial using a pre–post comparison design. The sample was derived in a three-stage cluster sampling procedure. Data collection for the endline surveys included a household survey and a facility-based survey. Women of childbearing age who gave birth at least once in the past 2 years prior to this survey and residing in the study area for more than 6 months were included in this study. Multilevel statistical techniques were used to examine individual and contextual effects related to health care demand and supply simultaneously and thus measure the relative contribution of the different levels to explaining factors associated with ANC use.

**Results:**

The working women were five times [odd ratio (OR): 5.41, 95% confidence intervals (CI) 4.36–6.70] more likely to report using ANC services than the women who were not working (OR: 5.41, 95% CI 4.36–6.70). Women living in a community with high poverty concentration were 32.0% (OR: 0.68, 95% CI 0.50–0.91) less likely to use ANC services than those in a community with low poverty concentration. Women living in a community with a medium concentration of women's modern contraceptive use were almost two times (OR: 1.88, 95% CI 1.70–2.12) more likely to use ANC services than those living in a community with a low concentration of women's modern contraceptive use. Women living in the health area where the level of ANC quality was high were three times (OR: 2.96, 95% CI 1.46–6.12) more likely to use ANC services than those in the health area where the ANC quality was low.

**Conclusion:**

Policies that increase the opportunity for improving the average ANC quality at the health facility (HF), the level of women's modern contraceptive use and women employment would likely be effective in increasing the frequency of use of antenatal services.

## Introduction

To achieve the sustainable development goals (SDGs), about two-thirds of sub-Saharan African countries will need to accelerate the reduction in maternal and under-five deaths (1, 2). In these countries, although much effort toward achieving the SDGs has focused on health systems and the supply side, including community case management of childhood illnesses (3–5), less attention has been paid to increasing demand for health care in general and antenatal care (ANC) in particular (1).

Antenatal care is one of the pillars of maternal and child health programs aimed at preventing and reducing maternal and child morbidity and mortality (6–9). The recognition of this fact has made ANC one of the main objectives of health programs, concerning the health needs of women during pregnancy (9–11). ANC has been shown to provide opportunities for the early detection of potential obstetric risks and, through counseling and education, motivates women to seek skilled assistance at birth (12–14). Thus, access to and adequate use of ANC services are essential to reduce maternal and neonatal mortality (6, 8). Improved maternal and neonatal outcomes have been associated with the utilization of ANC services (7, 12, 15, 16).

Like other African countries, Burkina Faso has a low utilization level of health care services in general and ANC services in particular (17). Indeed, despite considerable efforts by the government and its development partners to promote the use of health care services, the rate of utilization of ANC does not seem to be increasing significantly. Indeed, recent data showed that only a third of women (38.0%) received at least four ANC services in Burkina Faso, and 39.1% received it in their first trimester (17).

The causes of low use of health services in general and of ANCs in particular have been studied by several authors in Burkina Faso and elsewhere, often solely from the point of view of health care demand (18–25) or supply (11, 26–30). The effect of health care supply and demand, taken together in a single analysis, on the use of ANC services should be documented. This type of analysis would contribute to the explanation of the use of ANC by identifying the factors associated with the use of ANC among childbearing women, taking into account the effect of health care supply and demand. This would contribute to identifying relevant variables related to health supply or demand, necessary for better planning of future interventions.

This study aims to contribute to the results of previous studies by considering in a single analysis the factors affecting the supply and demand of health care in the analysis of the factors associated with the use of ANC.

## Materials and Methods

### Study Setting

The study was conducted in six administrative regions (Boucle du Mouhoun, Center-Est, Center-Nord, Center-Ouest, Nord, and Sud-Ouest) of Burkina Faso. The preliminary results of the fifth general population and housing census of Burkina Faso, carried out in 2019, indicated the total resident population of 20,487,979 inhabitants (31). According to the same source, the vast majority of the population (73.7%) lives in rural areas in 2019 (31).

According to the results of the demographic module of the continuous multisectoral survey carried out in 2019 in Burkina Faso, the vast majority of the population was affected by illiteracy (65.5% in 2014), and the poverty coverage rate at the national poverty line was estimated at 40.10% of the total population (32). The 2018 Human Development Index of the United Nations Development Program ranks Burkina Faso 182 out of 189 countries and territories with comparable data. According to the 2010 demography and health survey results for Burkina Faso, fewer than six in 10 women (58.4%) make two to three antenatal visits during pregnancy, and only three in 10 women (33.1%) make four or more visits during pregnancy (33).

### Data Source

To achieve the objectives of this study, two quantitative data sources were used: endline survey data (2017) for the impact evaluation of performance-based financing (PBF) in Burkina Faso and routine health facility (HF) data.

#### Endline Survey Data for the Impact Evaluation of PBF

The PBF impact evaluation was a blocked-by-region cluster random trial using a pre–post comparison design. The aim was to compare indicators between intervention areas (12 PBF intervention districts) and control areas (12 districts selected as controls) over a period before and after the intervention within the framework of the PBF impact evaluation. The sample was derived using a three-stage cluster sampling procedure, described in detail elsewhere (34).

Data collection for the endline surveys included a household survey and a facility-based survey. The household survey collected data on household characteristics and members: the health status of each member of the household and the use of health services, perception of the quality of services, ANC, postnatal care, immunization of children, and the use of the services of community health workers (CHW). The facility-based survey comprised different data collection tools with different data sources and respondents: HF records, providers' questionnaire, direct observations (curative consultations of under-five and antenatal consultations), exit interviews (curative consultations and antenatal consultations), and the CHW questionnaire. All HFs and all households included in this study responded to the questionnaires. This paper is based on the HF and household surveys.

#### Routine HF Data

Routine data from HFs (2017) were obtained from the General Directorate of Studies and Sector Statistics of the Ministry of Health. For health districts, numbers of resident population and HFs were provided. Data from the 24 health districts included in the performance-based financing (RBF) were extracted for analysis.

### Study Population and Sample Sizes

The source populations for this study were all permanent women residents in the study area who gave birth at least once in the last 2 years preceding the endline survey, irrespective of the outcome of delivery. Women of childbearing age who did not give birth at least once in the past 2 years prior to this survey and/or who resided in the study area for <6 months were excluded from this study. The analysis focuses on the responses from 8,174 women.

### Selected Variables

#### Outcome Variable

In this study, the dependent variable was the utilization of ANC services. This is a dichotomous variable (Yes/No), defined by whether or not the respondent used ANC services. This study classified the users of ANC services as women who have had at least four ANC visits for their pregnancies (<4 = 0 and ≥4 = 1). This classification was based on the reproductive health policy and standards of the Ministry of Health of Burkina Faso of a new model of ANC for women without complicated pregnancies (35).

#### Explanatory Variables

Analyzing the determinants of health care utilization is particularly challenging. This complexity comes first from a large number of factors that may affect the utilization of health care. In practice, all these data are never available for analysis, which limits the scope of the studies. With the multitude of studies describing the patterns of utilization in different health care settings, several scholars have developed explanatory frameworks that identify the predictors of health care utilization (36).

The variables used in the study are informed by the previous literature on the determinants of the utilization of ANC (18, 37–40). At individual and household levels, this study used mothers' age, the use of modern contraceptives, education, occupation, and household wealth index and size (Table 1).

**Table 1:** Measurement of explanatory variables included in the estimated models.

**Characteristics**	**Measurement**
Mother's age at last birth	The questionnaire did not include a direct question on the age at last birth; we computed this indicator by subtracting the child's age from the woman's current age and rounding the result to the nearest whole number.
Mother's use of modern contraceptive	We measure this indicator through reported use of modern contraceptive (Yes/No).
Mother's education	The highest level of education attained is divided into two categories: none and primary and above (combining primary and higher education categories together).
Mother's occupation	It has been re-coded in two categories: not working [Employee, employee, piecework (daily work), self-employed/self-employed worker, member of a producers' cooperative, family worker, apprentice] and working [looking for a job (unemployed), pupils/students, housewife].
Last birth order	We distinguish between mothers whose most recent birth is rank 1 and 2 and those whose most recent birth is of a higher rank. It has been re-coded first-order birth, second-order birth, third-order birth and above
Household wealth index^*^	We constructed a scale for household wealth index from information on possession of specific household items and utilities. The resulting scale was divided into five quintiles, coded poorest, poorer, middle, richer and richest.
Household size	It has been re-coded in 1–3, 4–5 and 6 and above.
Community concentration of female education	Aggregate values of community level of female education are measured by the proportion of women with a minimum of primary level of education derived from data on respondent's level of education categorized as: “0% = Low”, “ <25% = Medium” and “25% and above = High”
Community concentration of poverty	Aggregate values of community-level poverty are measured by the proportion of households in the poorest wealth quintile derived from data on wealth index categorized as: “ <15% = Low”, “15–40% = Medium” and “>40% = High”.
Community concentration of modern contraceptive prevalence	Aggregate values of community level of modern contraceptive prevalence are measured by the proportion of women who are currently using modern contraceptive categorized as: “ ≤ 15% = Low”, “15–40% = Medium” and “>40% = High”.
Type of place of residence	This variable was derived from the question on the type of place of residence. The variable place of residence recorded as rural and urban in the data set was retained without change.
Number of health facilities per 1,000 people	This information came from the routine health facility data.
The average quality of ANC at the facility	Data are captured by the facility survey from the exit interviews. The average quality of ANC at the facility, a composite quality index is constructed. The components considered, and demarcated as true/false, include the following: attendance by skilled health worker, weight and height measured, blood pressure checked, urine and blood sample taken, told about complications, given or bought iron tablets, and took fansidar as prophylaxis for malaria prevention (categorized as: “0% = Low”, “0–25% = Medium” and “>25% = High”.
Average distance between HF and households	It is computed from the geographic coordinates of households and health facilities (categorized as 1 = <1 km, 2 = 1–4 km and 3 = 5 km and above).
Performance-based financing^**^	It is categorized as 0 = control district and 1 = intervention district.

* *Described in more detail elsewhere (41)*.

** *Described in more detail elsewhere (34)*.

At the community level, variables often cited in the literature (37, 38, 42) capture characteristics such as accessibility, economic status, and other health system factors: community concentration of female education, community concentration of poverty, community concentration of modern contraceptive prevalence, and the type of place of residence.

For the health district/HF level, this study used HF density (number of HFs per 1,000 people), the average quality of ANC at the facility, and the average distance between HF and households and PBF.

In this study, the supply side is proxied by the density of HFs in the health district, the average distance between HF and households, and the average quality of ANC at the reference facility and PBF. The uptake of ANC indicates demand and distance to the nearest facility and captures the interaction between supply and demand.

### Statistical Analysis

Statistical analysis begun with cross-tabulations, and their statistical associations were used. The bivariate relationships indicated by the bivariate analysis may be due to interrelationships between the different characteristics measured as well as unmeasured characteristics at the community and health district level. Therefore, we used multilevel modeling to find the determinants of the use of ANC and analyze the variance of the use of ANC in its fixed and random components.

In the endline survey data for the impact evaluation of PBF in Burkina Faso, individual-level data followed a three-level hierarchical structure with the individual (women and household) at level 1 (*i*), nested within communities [or enumeration section (village)] at level 2 (*j*), and districts at level 3 (*k*). Multilevel statistical techniques provide a technically robust and efficient framework to account for the complex survey design and to assess variation in outcomes by multiple levels (43, 44). As suggested by its name, multilevel modeling enables simultaneous examination of the circumstances of individuals at one level in the context of multiple higher geographic, administrative, and social levels and thereby discerns the relative contribution of different levels to the scientific question of interest (43, 44).

For the binary outcome variable [ANC use (<4 = 0 and ≥4 = 1)], a series of three-level random intercept multilevel logistic regression models were estimated based on a logit link function.

The multilevel analysis began with an empty model to test the null hypothesis that the variance at the community and health district level in the use of ANC services is zero and to assess whether our data justify the decision to assess random effects at the community and health district level.

The null model with no predictor variable was run to serve as a baseline to compare changes in variance estimates in subsequent models (Model 0):


(1) $$\begin{array}{l} \text{Model }0:\text{logit}\left(\pi_{ijk}\right)=\;\beta_{0}+\left(f_{0k}+v_{0jk}\right), \end{array}$$

where β_0_ represents the median log odds of antenatal use across all study areas, and the bracketed terms in Model 0 represent the random effects associated with districts and communities, respectively. The term *f*_0k_ is a district-specific residual that represents a departure of each district from the national median log odds of antenatal use; *v*_0jk_ is a community-specific residual conditional on the district.

In subsequent models, all the individual-level covariates (*X*_*ijk*_) were included first in Model 1:


(2) $$\begin{array}{l} \text{Model }1:\text{logit}\left(\pi_{ijk}\right)=\;\beta_{0}+\text{β}X_{ijk}+\left(v_{0k}+u_{0jk}+e_{ijk}\right) \end{array}$$

Then, the variables related to community (Model 2) and district (Model 3) were added to evaluate the relative importance of the individual effect of the use of ANC versus contextual effect:


(3) $$\begin{array}{l} \begin{array}{c} \text{Model}2:\text{logit}(\pi_{ijk})=\beta_{0}+\text{β}X_{ijk}+\text{β}Y_{jk} \end{array}\\ \;\begin{array}{cc} + & \left(v_{0k}+u_{0jk}+e_{ijk}\right) \end{array} \end{array}$$

(4) $$\begin{array}{l} \begin{array}{c} \text{Model}3:\text{logit}(\pi_{ijk})=\beta_{0}+\text{β}X_{ijk}+\text{β}Y_{jk} \end{array}\\ \;\begin{array}{cc} + & \text{β}Z_{k}+(v_{0k}+u_{0jk}+e_{ijk}) \end{array} \end{array}$$

For each successive model, the proportion of variance in the log odds of the use of ANC explained by additional factors was computed by subtracting the variance of the model with the most terms from the variance of the simplest model and converting it to a percentage. Data were prepared, and all multilevel models were estimated using STATA 16.1. For interpretation, we report odd ratios (OR) and 95% confidence intervals (CI).

### Ethical Aspects

No constraints or restrictions are placed on the autonomy and independence of the study or the publication of its results. The Ethics Committee of the Medical Faculty of the University of Heidelberg (Protocol No. S-272/2013) and the Burkina Faso ethics committee for health research (Protocol No. 2013-7-06) have approved the study. Written consent was obtained from all respondents prior to the survey. The database was anonymized to ensure respondents' confidentiality.

## Results

Overall, among the respondents included in this study, it is noted that the proportion of women who used at least four ANC services during their last pregnancy was 80.80% in 2017.

### Bivariate Analysis

Tables 2, 3 present the variations in the indicator of the use of ANC services according to certain sociodemographic, household, community, HF, and health district factors. Table 1 shows the distribution of women who paid at least four ANC visits during their last pregnancy according to the individual and household's sociodemographic characteristics. There was a significant relationship (*p* < 0.01) between mother's age, mother's use of modern contraceptives, and the ANC utilization indicator. There was also a significant relationship between the ANC indicator and household size. The relationship between the ANC indicator and birth order, as well as the household wealth index and size, were not significant at the bivariate level.

**Table 2:** Variations in indicators of the use of antenatal care (ANC) services, by selected individual and household characteristics.

**Characteristics**	***n***	**≥4 Antenatal care visits (%)**	***X*** **^2^ probability**
**Mothers' age at last birth**			
15–19	551	80.94	0.03
20–24	1,688	80.15	
25–29	2,004	81.89	
30–34	1,717	80.84	
35–39	1,298	79.51	
40–44	654	76.91	
45–49	262	73.28	
**Mother's modern contraceptive use**			
Yes	2,434	81.88	0.039
No	5,740	80.35	
**Mother's education**			
No education	7,265	80.56	0.035
Primary and above	909	82.73	
**Mother's occupation**			
Not working	4,231	72.52	0.000
Working	3,943	89.78	
**Birth order**			
First birth	3,848	80.57	0.311
Second birth	3,704	80.74	
Third birth and above	622	82.74	
**Household wealth index**			
Poorest	1,368	82.09	0.581
Poorer	1,475	79.25	
Middle	1,592	80.15	
Richer	1,836	78.92	
Richest	1,903	80.82	
**Household size**			
1–3	750	61.07	0.114
4–5	1,817	55.75	
6 and above	5,607	43.64	
All respondents	8,174	80.80	

*Source: Authors' own calculations from endline (2017) survey data for the impact evaluation of performance-based financing (PBF) in Burkina Faso*.

**Table 3:** Variations in indicators of the use of ANC visits, by selected community and districts characteristics.

**Characteristics**	***N***	**≥4 Antenatal care visits (%)**	***X*** **^2^ probability**
**Community factors**			
**Type of place of residence**			
Rural	7,538	80.43	0.003
Urban	636	85.22	
**Community concentration of women education**			
Low (0%)	1,863	75.58	0.000
Medium (>25%)	5,403	82.45	
High (25% and above)	908	81.72	
**Community concentration of poverty**			
Low (<15%)	2,245	87.04	0.000
Medium (15–40%)	4,134	75.06	
High (40% and above)	1,795	86.24	
**Community concentration of women's modern contraceptive use**			
Low (<15%)	1,573	76.10	0.000
Medium (15–40%)	5,781	80.71	
High (40% &+)	820	90.49	
**Health district and HF factors**			
**Number of health facilities per 1,000 inhabitants**			
Low (<0.15)	2,411	68.89	0.000
Medium (0.15–0.2)	3,586	82.40	
High (0.20 and above)	2,177	91.73	
**Average distance between HF and households**			
>1 km	1,107	88.17	0.000
1–4 km	3,438	81.04	
5 km and above	3,629	78.45	
**Average quality of ANC at the facility**			
Low	6,315	49.24	0.000
Medium	1,255	86.05	
High	604	92.38	
**PBF**			
Control	1,899	80.75	0.520
Intervention	6,275	81.41	
All respondents	8,174	80.80	

*Source: Authors' own calculations from endline (2017) survey data for the impact evaluation of PBF in Burkina Faso*.

Regarding the characteristics of the community, the HF, and the health district (Table 2), there was a significant relationship between the type of place of residence, community concentration of women education, community concentration of poverty, community concentration of women's use of modern contraceptives, the average distance between HF and households, the average quality of ANC at the facility, and the number of HFs per 1,000 inhabitants with the ANC indicator. PBF was not significantly associated with the ANC indicator.

### Multilevel Models

The results presented in Table 4 show that for the ANC service utilization indicator, there was considerable heterogeneity between communities and health districts. For example, the variance at the health district level in the empty model is large and significant, indicating considerable differences in the use of ANC and health districts. The intraclass correlation (ICC) in the empty model indicates that 46.3% of the total variance in the use of ANC services is attributable to the differences between the health districts.

**Table 4:** Parameter coefficients for the multilevel model of the use of ANC visits—assesses the role of variables at different levels.

**Characteristics**	***n***	**Model 0**	**Model 1 (OR CI 95%)**	**Model 2 (OR CI 95%)**	**Model 3 (OR CI 95%)**
**Fixed effects**					
**Mother's age**					
15–19	551		1.00	1.00	1.00
20–24	1,688		1.18 (0.82–1.70)	1.17 (082–1.70)	1.18 (0.82–1.70)
25–29	2,004		1.05 (0.72–1.51)	1.04 (0.73–1.52)	1.05 (0.72–1.52)
30–34	1,717		1.04 (071–1.52)	1.05 (0.71–1.54)	1.04 (0.71–1.54)
35–39	1,298		0.75 (0.51–1.12)	0.76 (0.52–1.13)	0.76 (0.52–1.130)
40–44	654		0.98 (0.64–1.52)	1.01(0.65–1.56)	1.01 (065–1.56)
45–49	262		1.29^*^ (0.74–2.24)	1.28^*^ (0.73–2.28)	1.27 (0.73–2.22)
**Mother's modern contraceptive use**					
Yes	2,434		1.00	1.00	1.00
No	5,74		0.96 (0.81–1.14)	0.95 (0.80–1.13)	0.87 (0.80–1.13)
**Mother's education**					
No education	7,265		1.00	1.00	1.00
Primary and above	909		1.95 (1.29–2.35)	1.54 (1.28–2.02)	1.53 (1.29–2.01)
**Mother's occupation**					
Not working	4,251		1.00	1.00	1.00
Working	3,923		5.50^***^ (4.48–6.76)	5.38^***^ (4.34–6.67)	5.41^***^ (4.36–6.70)
**Birth order**					
First birth	3,896		1.00	1.00	1.00
Second child	3,687		1.02^*^ (0.71–1.99)	1.01 (0.71–1.31)	1.01 (0.86–1.43)
Fourth child and above	591		1.03 (0.67–1.78)	1.02 (0.67–1.28)	1.02 (0.75–1.36)
**Household wealth index**					
Poorest	1,368		1.00	1.00	1.00
Poorer	1,475		1.048^*^ (0.60–1.52)	1.05 (0.60–1.56)	1.06 (0.59–1.29)
Middle	1,592		1.063^*^ (0.73–1.54)	1.07 (0.74–1.51)	1.07 (074–1.32)
Richer	1,836		1.174^*^ (0.64–1.47)	1.18 (0.63–1.43)	1.18 (0.62–1.49)
Richest	1,903		1.126 (0.66–1.42)	1.14 (0.66–1.46)	1.13 (0.66–1.47)
**Household size**					
1–3	750		1.00	1.00	1.00
4–5	1,817		1.013 (065–1.29)	1.02 (0.65–1.64)	1.025 (0.65–1.65)
6 and above	5,607		0.87 (063–1.22)	0.86 (0.62–1.45)	0.88 (0.62–01.21)
**Community factors**					
**Type of place of residence**					
Rural	7,538			1.00	1.00
Urban	636			1.12 (0.62–1.47)	1.012 (0.61–1.56)
**Community concentration of women education**					
Low (0)	1,863			1.00	1.00
Medium (0–25%)	5,403			1.93 (1.75–2.12)	1.92 (1.74–2.11)
High (25% and above)	908			1.99 (1.67–2.22)	2.04 (1.66–2.2.36)
**Community concentration of poverty**					
Low (<15%)	2,245			1.00	1.00
Moderate (15–40%)	4,134			0.61^***^ (0.47–0.80)	0.62^***^ (0.46–0.81)
High (40% and above)	1,795			0.67^***^ (0.49–0.90)	0.68^***^ (0.50–0.91)
**Community concentration of women modern contraceptive use**					
Low (<15%)	1,573			1.00	1.00
Moderate (15–40%)	5,781			1.88^**^ (1.70–2.12)	1.88^**^ (1.70–2.12)
High (40% and above)	820			2.76 (2.19–3.60)	2.41(2.20–3.60)
**District and health facilities factors**					
**Number of health facilities per 1,000 inhabitants**					
Low (<0.15)	2,411				1.00
Medium (0.15–0.2)	3,586				1.43 (1.09–2.03)
High (0.20 and above)	2,177				1.38 (1.06–2.67)
**Average distance between HF and households**					
Low	1,273				1.00
Medium	4,320				0.96 (0.54–1.17)
High	2,581				0.91 (0.36–2.11)
**The average quality of ANC at the health facility**					
Low	6,315				1.00
Medium	1,255				1.19 (1.02–2.26)
High	604				2.96^**^ (1.46–6.12)
**PBF**					
*Control district*	1,899				*1.00*
Intervention district	6,275				1.912 (1.30–3.01)
**Random effects**					
District-level variance		1.828^***^ (0.540)	2.241^***^ (0.670)	2.167^***^ (0.671)	1.956^***^ (0.614)
Residual intraclass correlation		0.463	0.402	0.401	0.378
Log likelihood		−2,634.787	−2,427.2153	−2,414.8049	−2,413.429
Akaike crit. (AIC)		5,275.575	4,894.431	4,883.610	4,896.251

*** *p < 0.01,*

** *p < 0.05,*

* *p < 0.1*.

*Source: Authors' own calculations from endline (2017) survey data for the impact evaluation of PBF in Burkina Faso*.

Table 4 shows the results of the saturated model, which assesses the role of variables at different levels. The most significant individual characteristic of the use of ANC services was the mother's occupation. The odds of reporting the use of ANC services increase with whether the mother is working or not, such that working women were five times more likely to report the use of ANC services than women who were not working.

Two of the community-level variables included in the model are found to be associated with the use of ANC services. These were the community concentration of poverty and women's use of modern contraceptives. Women living in areas with a low concentration of poverty have higher odds of receiving four or more ANC visits as opposed to women living in communities with a medium or high concentration of poverty. For example, women living in a community with medium poverty concentration were 33.30% less likely to use ANC services than those living in a community with a low poverty concentration. Women living in a community with a medium concentration of women's use of modern contraceptives were two times more likely to use ANC services than those living in a community with a low concentration of women's use of modern contraceptives.

The most significant health district and HF level characteristic of the use of ANC services was the average quality of ANC at the HF. Women living in the health area where the level of ANC quality was high were four times more likely to use ANC services than those living in the health area where the ANC quality was low.

Finally, the residual ICC remains noticeably high, indicating that even after considering individual, household and community factors, there remained considerable consolidation in the use of ANC services at the health district level.

## Discussion

The objective of this study was to identify the different factors that may influence the use of ANC services by pregnant women in Burkina Faso, by considering factors related to the health care supply and demand jointly in a single analysis. The results of this study support the conceptual framework that guided this study. The findings demonstrate the influence of health care supply and demand factors on the use of ANC in Burkina Faso.

Regarding factors related to health care demand, there was a significant relationship between the use of ANC services and the mother's occupation at the individual level. The community concentration of poverty and women's use of modern contraceptives were found to be associated with the use of ANC services. Regarding factors linked to health care supply, the average quality of ANC at the HF was found to be associated with the use of ANC services.

At the individual level, the use of ANC improved with higher mother's occupation. Mother's working status appeared as one variable with a very strong influence on the use of ANC services. The results of this study showed that the working mother was more likely to make antenatal visits many times than unemployed mothers. This finding is consistent with that by Assefa and Tadesse (45), Tawiah (46), and Sharma et al. (47). Mothers' working status was related to the social environment. Working pregnant women have a wider social environment than housewives. This allowed pregnant women to access more information related to pregnancy, including ANC. The influence of information from co-workers and other information that pregnant women got during work increased pregnancy knowledge that would eventually initiate ANC visits according to recommendations (48).

At the community level, the lower the concentration of poverty, the higher the propensity of getting four or more antennal care visits. This finding is consistent with the outcomes of studies conducted in Nigeria (49, 50). The community with a high concentration of women's use of modern contraceptives exerted strong effects on the use of ANC services. This finding is consistent with the finding from studies conducted elsewhere (51, 52). The higher the average quality of ANC at the HF, the higher the propensity of getting four or more antennal care visits. This finding is consistent with that from the two studies conducted in Zambia by Chama-Chiliba and Koch (38) and Kyei et al. (53).

In summary, there is evidence from this study that the use of ANC services is determined not only by the observed factors related to individual characteristics, community, and health district but also by the unobserved effects at the individual, community, and health district level. Estimates of the ICC in using ANC services remained considerable, even after controlling for selected variables at the individual, community, and health district level. Large residuals in the health district variance in the use of ANC suggest that there are other factors at the individual, community, and health district level that are not accounted for in this analysis. The significant health district-level random effects that our study found demonstrate the need to contextualize efforts aimed at promoting the use of maternal services. There are obviously some unmeasured factors at the health district level that predict the use of ANC services. Possible unobserved factors could include cultures and customs in pregnancy and birthing care that were not measured in this study that may help explain part of the health district variance.

### Limitations of This Study

This study used data from the endline survey for the PBF impact evaluation, which uses three-stage cluster sampling procedures to ensure reliability and a multistage probability sampling methodology to select clusters and households from geographic sampling frames that cover the study area. However, there are some limitations to this study. The first limitation is the cross-sectional nature of the data collected, which means the outcome and explanatory variables were measured simultaneously, and therefore cannot guarantee any causality of associations. The second limitation is the recall and self-reporting bias of the information collected by the endline survey for the PBF impact evaluation. However, to reduce as much as possible various limitations and possible biases of this study, descriptive and multivariate analysis methods were combined.

## Conclusion

Several factors influencing the use of ANC services occur at different levels: individual/household, community, and health district. At the individual household level, the mother's occupation was consistently a strong predictor of the use of ANC services considered in this study. At the community level, living in a community with a low concentration of poverty or in a community with a high concentration of women using modern contraceptive methods were associated with the use of ANC services. Women living in the health area where the level of ANC quality was high were four times more likely to use ANC services than those living in the health area where the ANC quality was low. To be optimally effective, interventions to promote the use of ANC services need to consider these findings.

The study findings have important implications for the design of health policy concerning maternal health in Burkina Faso. Policies that increase the opportunity to improve the average ANC quality at the HF, the level of women's use of modern contraceptive methods, and women employment would likely be effective in increasing the frequency of use of ANC services. Consequently, government policies should target economically disadvantaged women so that the frequency of antenatal visits can be increased.

## Data Availability Statement

The raw data supporting the conclusions of this article will be made available by the authors, without undue reservation.

## Ethics Statement

The studies involving human participants were reviewed and approved by the Ethics Committee of the Medical Faculty of the University of Heidelberg (Protocol Number S-272/2013); Burkina Faso National Ethics Committee (Protocol Number 2013-7-06). Written informed consent to participate in this study was provided by the participants' legal guardian/next of kin.

## Author Contributions

HB, AB, HH, and MD developed the detailed plans for the fieldwork, designed the data collection instruments, implemented, and supervised the fieldwork. HB and AS conceived and designed this paper and developed the analysis strategy. HB analyzed the data and wrote the first draft. AS is the overall guarantor author. All authors reviewed, provided inputs, and approved the final paper.

## Funding

The baseline (2013) and endline (2017) survey for the impact evaluation of PBF in Burkina Faso was supported by the World Bank through the Health Results Innovation Trust Fund (HRITF).

## Conflict of Interest

The authors declare that the research was conducted in the absence of any commercial or financial relationships that could be construed as a potential conflict of interest.

## Publisher's Note

All claims expressed in this article are solely those of the authors and do not necessarily represent those of their affiliated organizations, or those of the publisher, the editors and the reviewers. Any product that may be evaluated in this article, or claim that may be made by its manufacturer, is not guaranteed or endorsed by the publisher.
